# In vitro activities of omadacycline, eravacycline, cefiderocol, apramycin, and comparator antibiotics against *Acinetobacter baumannii* causing bloodstream infections in Greece, 2020–2021: a multicenter study

**DOI:** 10.1007/s10096-023-04616-7

**Published:** 2023-05-03

**Authors:** Irene Galani, Vassiliki Papoutsaki, Ilias Karaiskos, Nikolaos Moustakas, Lamprini Galani, Sofia Maraki, Viktoria Eirini Mavromanolaki, Olga Legga, Kimon Fountoulis, Evangelia D. Platsouka, Panagiota Giannopoulou, Helen Papadogeorgaki, Maria Damala, Efrosini Chinou, Aggeliki Pasxali, Ioannis Deliolanis, Helen Vagiakou, Efthymia Petinaki, Anastasia Chli, Eleni Vagdatli, Polyzo Kazila, Vassiliki Papaioannou, Konstantina Kontopoulou, Atalia Noemi Ferke, Eleni Moraitou, Anastasia Antoniadou, Helen Giamarellou

**Affiliations:** 1 Infectious Diseases Laboratory, 4th Department of Internal Medicine, National and Kapodistrian University of Athens, School of Medicine, Athens, Greece; 2 Microbiology Laboratory, Hygeia General Hospital, Athens, Greece; 3 1st Department of Internal Medicine-Infectious Diseases, Hygeia General Hospital, Athens, Greece; 4 Department of Clinical Bacteriology, Parasitology, Zoonoses and Geographical Medicine, University Hospital of Heraklion, Heraklion, Greece; 5 Department of Microbiology, General Hospital of Lamia, Lamia, Greece; 6 Department of Clinical Microbiology, Evangelismos General Hospital, Athens, Greece; 7 Department of Microbiology, General Hospital of Nea Ionia, “Konstantopouleio-Patission”, Athens, Greece; 8 Department of Microbiology, Thriasio General Hospital of Elefsina, Elefsína, Greece; 9 Microbiology Department, “Alexandra” General Hospital of Athens, Athens, Greece; 10 Department of Microbiology, St Savvas Cancer Hospital, Athens, Greece; 11 Microbiology Laboratory, General Hospital of Corfu, Corfu, Greece; 12 Department of Microbiology, Laiko General Hospital, Athens, Greece; 13 Microbiology Laboratory, General Hospital of Athens “G. Gennimatas”, Athens, Greece; 14 Department of Microbiology, University Hospital of Larissa, Larissa, Greece; 15 Microbiology Laboratory, General Hospital of Kavala, Kavala, Greece; 16 Microbiology Department, Hippokration General Hospital, Thessaloniki, Greece; 17 Department of Clinical Chemistry, “THEAGENEIO” Cancer Hospital, Thessaloniki, Greece; 18 Microbiology Department, KAT Hospital, Athens, Greece; 19 Department of Microbiology, General Hospital of Thessaloniki “G. Gennimatas”, Thessaloniki, Greece; 20 Department of Microbiology, General Hospital of Rhodes, Rhodes, Greece; 21 Department of Clinical Microbiology, Sotiria General Hospital of Chest Diseases, Athens, Greece

**Keywords:** Acinetobacter baumannii, OXA-23, ArmA, IC II, Apramycin, Cefiderocol, Eravacycline

## Abstract

Resistance of *Acinetobacter baumannii* to multiple clinically important antimicrobials has increased to very high rates in Greece, rendering most of them obsolete. The aim of this study was to determine the molecular epidemiology and susceptibilities of *A. baumannii* isolates collected from different hospitals across Greece. Single-patient *A. baumannii* strains isolated from blood cultures (*n* = 271), from 19 hospitals, in a 6-month period (November 2020–April 2021) were subjected to minimum inhibitory concentration determination and molecular testing for carbapenemase, 16S rRNA methyltransferase and *mcr* gene detection and epidemiological evaluation. 98.9% of all isolates produced carbapenemase OXA-23. The vast majority (91.8%) of OXA-23 producers harbored the *armA* and were assigned mainly (94.3%) to sequence group G1, corresponding to IC II. Apramycin (EBL-1003) was the most active agent inhibiting 100% of the isolates at ≤16 mg/L, followed by cefiderocol which was active against at least 86% of them. Minocycline, colistin and ampicillin-sulbactam exhibited only sparse activity (S <19%), while eravacycline was 8- and 2-fold more active than minocycline and tigecycline respectively, by comparison of their MIC_50_/_90_ values. OXA-23-ArmA producing *A. baumannii* of international clone II appears to be the prevailing epidemiological type of this organism in Greece. Cefiderocol could provide a useful alternative for difficult to treat Gram-negative infections, while apramycin (EBL-1003), the structurally unique aminoglycoside currently in clinical development, may represent a highly promising agent against multi-drug resistant *A. baumanni* infections, due to its high susceptibility rates and low toxicity.

**Supplementary Information:**

The online version contains supplementary material available at 10.1007/s10096-023-04616-7.

## Introduction

*Acinetobacter baumannii* is an important nosocomial pathogen causing severe infections, particularly in intensive care units. It is also known for its ability to acquire resistance to several antimicrobial agents [1, 2]. The most common infections in clinical settings are bloodstream infections (BSI) including catheter-related BSI (CRBSI) and hospital acquired and ventilator associated pneumonia [3]. Recent data from the European Antimicrobial Resistance Surveillance Network (EARS-Net) show a large and statistically significant increase of *Acinetobacter* spp. BSIs in the European Union (EU) and European Economic Area (EEA) during 2020–2021 (+ 57% compared to 2018-2019), a period which represents the first years of the COVID-19 pandemic [4]. In a systemic review of pan-drug-resistant (PDR) Gram-negative bacteria epidemiology and prognosis, *Pseudomonas aeruginosa* and *A. baumannii* were the most common PDR reported species (33%), followed by *Klebsiella pneumoniae* (24%) [5]. PDR infections were associated with excess mortality, mounting up to 71% regardless of the infection source [5].

There is no optimal therapeutic strategy for the management of extensively drug-resistant (XDR) *A. baumannii* infections. Sulbactam, meropenem, minocycline, tigecycline and polymyxins, have served as last-resort antibiotics against infections in the critically ill over the last decades [6, 7]. Cefiderocol and eravacycline, two new antimicrobial agents with in vitro susceptibility against *A. baumannii*, have been approved by the US Food and Drug Administration (FDA) in 2019 and 2018 and by the European Medicines Agency (EMA) in 2020 and 2018 respectively. The major problem is their limited commercial availability. Εravacycline is unavailable in Europe and cefiderocol has only been recently launched in a few European countries (United Kingdom, Germany and Italy), mostly serving compassionate use purposes [8].

Apramycin has been proposed as a possible next-generation aminoglycoside, and it is currently the only new aminoglycoside in clinical development (Phase I) [9, 10]. EBL-1003, a crystalline free base of apramycin, is a candidate drug that recently completed the first-in-human study to assess the safety tolerability and general pharmacodynamic profile of the drug. Unpublished data shows that EBL-1003 is both safe and well tolerated and the Juvadis team are planning a further phase I trial in patients with complicated urinary tract infections - one of the disease areas where EBL-1003 seems most promising [11, 12]. Based on its unique chemical structure, comprising an unusual bicyclic octose moiety, apramycin (EBL-1003) evades almost all clinically relevant aminoglycoside modifying enzymes (AMEs) and is also unaffected by 16S rRNA-methyltransferase (RMTase) -mediated pan-aminoglycoside resistance [13].

In Greece, in a multicenter study conducted between 2010 and 2015, resistance of *A. baumanii* to multiple clinically important antimicrobials was found to have increased to very high rates, rendering most of antimicrobials in clinical use, obsolete [14]. According to the European Antimicrobial Resistance Surveillance Network (EARS-Net), combined resistance to fluoroquinolones, aminoglycosides and carbapenems resistance in *Acinetobacter* species from invasive infections in Greece varied between 84.0% in 2016 and 90.8% in 2020 [15]. Additionally, the ECDC reported that countries with ≥ 50% carbapenem resistance in *Acinetobacter* spp. in 2018–2019, experienced the most noticeable increases (+116%) in *Acinetobacter* spp. BSIs in 2020–2021 compared with 2018–2019 and suggested surveillance at local, national and EU/EEA levels to monitor whether this worrying development is halted or even reversed [4].

We therefore undertook this study so as to analyse the resistance phenotypes, the carbapenemase and aminoglycoside modifying gene content and the evolution of clonal lineages among *A. baumannii* blood isolates recovered from Greek hospitalized patients during 2020–2021. Furthermore, we evaluated the in vitro activities of older along with newer agents such as cefiderocol, apramycin (EBL 1003) and the more advanced tetracyclines omadacycline and eravacycline.

## Material and methods

### Bacterial strains

**Fig. 1:**
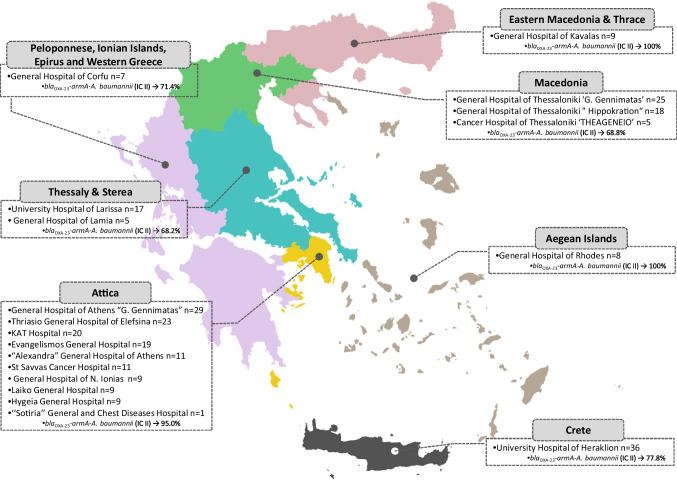
Geographical map showing the location of the19 participating hospitals providing *Acinetobacter baumannii* blood isolates, Greece, 2020–2021, and the percentage of the prevailing clone

All *A. baumannii* strains included in the study were consecutive, single-patient clinical isolates provided by the microbiology laboratories of 19 participating hospitals located in all seven Health Districts of Greece (Fig. 1). These were collected in a 6-month period (November 2020–April 2021) and originated from distinct blood infection cases. The isolate recovered first from each case was only included. A total of 271 blood isolates were studied. Each hospital contributed a median number of 11 (min 1, max 36) isolates.

Data on the source and the date of isolation as well as the initial susceptibility results at the local laboratories were also provided. All isolates were re-submitted for susceptibility testing using the VITEK2 system (bioMerieux, Marcy-l’Etoile, France) and kept frozen at −80°C until the day of further testing.

### Antimicrobial susceptibility

Initial susceptibilities of the isolates to ampicillin/sulbactam, ciprofloxacin, levofloxacin, gentamicin (GM), amikacin (AMK), tobramycin (TOB), tigecycline (TGC) and trimethoprim/sulfamethoxazole (SXT) were determined using the VITEK 2 system (bioMerieux, Marcy-l’Etoile, France).

Minimum inhibitory concentrations (MICs) of imipenem, meropenem, minocycline, GM, AMK, TOB, apramycin (EBL-1003), SXT and colistin were determined by the broth microdilution method [16]. Liofilchem MIC Test Strips (Liofilchem S.r.l., Roseto degli, Abruzzi, Italy) were used for MIC determinations of eravacycline, omadacycline and TGC, while disk diffusion was performed for testing cefiderocol (Cefiderocol 30μg Disc, Liofilchem S.r.l.) as recommended by the European Committee on Antimicrobial Susceptibility Testing (EUCAST), due to problems in accuracy and reproducibility of all MIC commercially available tests [17]. *Escherichia coli* ATCC 25922 and *Pseudomonas aeruginosa* ATCC 27853 were used as quality control (QC) strains. Results were considered valid if both QC strains tested in each experiment fell within the Clinical and Laboratory Standards Institute (CLSI) designated QC MIC ranges or were within +/-1 mm of the disk diffusion EUCAST target values (27 mm for ATCC 25922 and 26 mm for ATCC 2785) [18, 19]. *E. coli* NCTC 13846 (*mcr-1*-positive) was additionally used as a third QC strain for colistin MIC determinations. Results were interpreted according to the CLSI and EUCAST recommendations [18, 20]. For tigecycline, eravacycline and omadacycline, MIC_50_ and MIC_90_ values were used as a form of susceptibility interpretive tool, due to lack of CLSI/EUCAST interpretive criteria for *Acinetobacter* spp. For apramycin (EBL-1003), the preliminary epidemiological cutoff value of 16 mg/L proposed by Juhas et al, was applied, as there are no established breakpoints for *A. baumannii* [13]. The acceptable MIC QC range for apramycin was set to a modal value of 4 mg/L for *E. coli* ATCC 25922 and an acceptable range of 2–8 mg/L [13]. All isolates were subcultured twice before testing. Multidrug-resistant (MDR), XDR and PDR strains were characterized as per criteria described by ECDC [21].

### Detection of antimicrobial resistance genes

Genes encoding common class D carbapenemases (*bla*_OXA-51_-like, *bla*_OXA-58_-like, *bla*_OXA-23_-like, *bla*_OXA-40_-like, *bla*_OXA-143_-like and *bla*_OXA-235_-like) and genes encoding for RMTs were detected by multiplex PCR protocols with specific primers and conditions (Supplementary Table S1). The presence of a*rmA* gene was further confirmed by simplex PCR with specific primers (Suppl. Table S1). Genes encoding class B metallo-β-lactamases (*bla*_IMP_, *bla*_VIM_, and *bla*_NDM_) were detected by simplex PCR with primers and conditions listed in Suppl. Table S1. All RMT-negative isolates (n=23) were screened for the presence of AME genes (*aph(3′)-VI*, *aac(6′)-Ib*, *aac(3′)-Ia*, *aac(3′)-IV*, *ant(2′)-Ia*), along with a subset of randomly selected RMT-positive isolates (n=55), by simplex ‘in-house’ PCR assays with specific primers (Suppl. Table S1). The presence of plasmid-mediated colistin resistance genes was screened by a multiplex PCR protocol optimized at the Danish National Food Institute (Kgs Lyngby, Denmark) (Suppl. Table S1). Template DNA was extracted from bacteria grown in Luria Bertani broth for 18 hr by using the PureLink^TM^ Genomic DNA Mini Kit (LifeTechnologies, Invitrogen^TM^, Carlsbad, CA, USA).

### PCR-based sequence group typing

Two trilocus multiplex PCRs (Suppl. Table S1), which selectively amplify Group 1 and Group 2 alleles of the *ompA*, *csuE*, and *bla*_OXA-51_-like, were used to assign the sequence groups and the corresponding major international clones IC I - IC III according to Turton et al [22]. Using this scheme, additional groups (G4, G5, G6, G7 and G11) have been defined according to new combinations of the PCR amplicons [23–25].

## Results and discussion

Susceptibilities of isolates interpreted according to CLSI and EUCAST breakpoints are shown in Table 1 and MIC and “cumulative percentage inhibited” distributions are presented in Suppl. Table S2. Two hundred sixty-nine (99.3%) isolates exhibited an MDR phenotype, with 265 (97.8%) of them to be defined as XDR and 149 (55.0%) as PDR isolates.

**Table 1:** Percentage of susceptibility to antimicrobial agents according to CLSI and EUCAST clinical breakpoints

	Susceptibility %		MIC Range mg/L	MIC_50_/MIC_90_ mg/L
	According to CLSI breakpoints	According to EUCAST breakpoints		
*β-Lactam combination agents*				
Ampicillin-sulbactam	11.8	IE		
Piperacillin-tazobactam	5.65	IE		
*Cephems*				
Cefiderocol	93.4	86.0*		
*Carbapenems*				
Imipenem	5.9	5.9		
Meropenem	1.1	1.1		
*Lipopeptides*				
Colistin	15.5^#^	15.5^^^		
*Aminoglycosides*				
Amikacin	1.5	1.5		
Gentamicin	1.1	1.1		
Tobramycin	4.8	4.8		
Apramycin (EBL-1003)	-	-	2–16	4/8
*Tetracyclines*				
Minocycline	18.8	IE		
Tetracycline	0.7	-		
Tigecycline	-	IE	0.12–16	4/8
Eravacycline	-	-	0.06–>32	2/4
Omadacycline	-	-	0.12–>32	8/>32
*Quinolones*				
Ciprofloxacin	0.7	ND		
Levofloxacin	0.7	0.7		
*Folate pathway antagonists*				
Trimethoprim-sulfamethoxazole	1.85	1.85		

IE, breakpoints not defined due to insufficient evidence; -, breakpoints not defined; *ND*, not determined

^*^Isolates exhibiting cefiderocol 30μg disk zone diameter ≥17mm which corresponds to MIC values below the PK-PD breakpoint of S ≤ 2mg/L

^#^Isolates not resistant to colistin (according to CLSI)

^^^Isolates assigned as wild type isolates without acquired resistance mechanisms (according to EUCAST)

Except for the intrinsic *bla*_OXA-51_-like gene, which was confirmed in all (100%) isolates, other forms of carbapenemase production were also confirmed for 268 (98.9%) isolates. All carried a *bla*_OXA-23_-like and four isolates additionally carried the *bla*_NDM_ (Table 2). The presence of an RMT-coding gene was confirmed in 246 (90.8%) isolates, all carrying the *armA* and exhibiting high-level resistance (MIC ≥ 256 mg/L) to amikacin, gentamicin and tobramycin. Additionally, 162 (59.8%) of the isolates carried a *bla*_TEM_. Isolates without the *armA harbored* mainly the *aph(3′)-VI* (75.0%) conferring resistance to amikacin and the *aac(3)-I* (37.5%) conferring resistance to gentamicin. Isolates resistant to tobramycin (16.7%) harbored the *aac(6′)-Ib.* Among isolates with an *armA,* although not all tested*, aph(3′)-VI* (90.1%) was the predominant AME gene, followed by *aac(6′)-Ib* (27.2%) and *aac(3)-I* (9.1%).

**Table 2:** Epidemiological and genotypic features of *Acinetobacter baumannii* strains included in the study

Sequence Group/Clonal lineages	Total No / Rate (%)	G1/IC II	G2/IC I	G4/IC II	G5	G6	G7	G11/IC I	UN*
*bla*_OXA-51_- harboring	3 / 1.1	1					1		1
* bla*_OXA-51_	2	1							1
* bla*_OXA-51,_ *bla*_TEM_	1						1		
*bla*_OXA-51,_ *bla*_OXA-23_ - harboring	264 / 97.4	238	4	2	6	11	1	1	1
* bla*_OXA-51,_ *bla*_OXA-23_	12	4	1	2	1	3	1	1	
* bla*_OXA-51,_ *bla*_OXA-23_, *armA*	83	75			2	5			1
* bla*_OXA-51,_ *bla*_OXA-23_, *armA, bla*_TEM_	161	155	1		2	3			
* bla*_OXA-51,_ *bla*_OXA-23_, *bla*_TEM_	7	4	2		1				
*bla*_OXA-51,_ *bla*_OXA-23_, *bla*_NDM_- harboring	4 / 1.5	2	1		1				
* bla*_OXA-51,_ *bla*_OXA-23_, *bla*_NDM_	2		1		1				
* bla*_OXA-51,_ *bla*_OXA-23_, *bla*_NDM_, *armA*	1	1							
* bla*_OXA-51,_ *bla*_OXA-23_, *bla*_NDM_, *armA, bla*_TEM_	1	1							
Total No/Rate (%)		241/88.9	5/1.8	2/0.7	7/2.6	11/4.1	2/0.7	1/0.4	2/0.7

^*^ UN, untypeable

The vast majority (n=241; 88.9%) of *A. baumannii* isolates were assigned to sequence group G1 corresponding to IC II (Table 2). Five isolates (1.8%) were assigned to G2 corresponding to IC I (Table 2). Two isolates from the same hospital were assigned to G4 and one isolate to G11, with their PCR-based band pattern differing from that of IC II or of IC I respectively, only by the absence of the *csuE* allele, which could simply be due to a single polymorphism in the primer annealing regions (Table 2). Seven, eleven and two isolates from 4, 3, and 2 hospitals respectively, were assigned to G5, G6 and G7 sequence groups, showing mixed combinations of amplicons in the two trilocus multiplex PCRs (Table 2). The *bla*_OXA-23_ was found in isolates of all sequence groups (Table 2), while isolates co-harboring *bla*_NDM_ (*n*=4) were polyclonal as two were assigned to G1 (IC II) (also harbored the *armA*), one in G2 (IC I) and one in G5 (Table 2) and were isolated in 4 different hospitals, two in Athens and two in Thessaloniki.

This study highlights the dissemination of XDR/PDR *bla*_OXA-23_-*armA*-harboring *A. baumannii* isolates, corresponding to IC II (87.8%), in Greek hospitals. OXA-23-producing carbapenem resistant *A. baumannii* (CR*Ab*) were first described in our country in 2010 [26]. According to published data, CR*Ab* isolates collected in Greece during 2015 belonged mainly to IC II and produced OXA-23 almost uniformly, whereas similar collections prior 2004 revealed predominance of the IC I clone and the OXA-58 carbapenemase [27, 28]. *A. baumannii* isolates shown to carry the *armA* gene were first recovered in 2003 in South Korea [29], and since then this gene has been reported in strains from China, Vietnam, Japan, North America, Norway, Italy, Bulgaria, Iran, and Algeria [30]. ArmA methylates the N7 position of nucleotide G1405 in 16S rRNA and confers high-level resistance to all widely used aminoglycosides (4,6-disubstituted deoxystreptamines), including plazomicin, which is the agent most recently introduced in clinical practice [13]. The association between IC II and *armA* was first reported in Greece in 2020 and described for CR*Ab* isolates recovered from five hospitals within the Athens metropolitan area during 2015-2016 [31]. The *armA* is always located on a functional composite transposon *Tn*1548, and it is often now reported among OXA-23-producing *A. baumannii* strains. However, the two resistance genes are not physically linked on a single plasmid [30]. Wherever multilocus sequence typing data are available, most OXA-23-ArmA positive *A. baumannii* isolates were identified to belong to ST2 and are consequently members of IC II [32].

There are very few antimicrobial agents in the market that retain activity against CR*Ab*, including polymyxins (colistin), aminoglycosides and tetracyclines (such as tigecycline and minocycline), limited by suboptimal pharmacokinetic characteristics, emergence of resistance, and/or toxicity [33]. This study confirmed that the most active antimicrobial in clinical use was minocycline, with 18.8% of the isolates exhibiting an MIC of ≤ 4mg/L, which is the CLSI susceptibility breakpoint. Only 15.5% of the isolates exhibited a colistin MIC of ≤ 2mg/L and were assigned as wild type isolates without acquired resistance mechanisms (Table 1, Suppl. Table S2). The alarmingly high resistance rates observed for colistin (84.5%) and minocycline (81.2%) in this study might be related to the isolate source (blood) and the predominance of IC II [34]. Both assets, according to Petropoulou et al., showed generally more resistant profiles compared to non-blood and IC I isolates, in a previous national collection of carbapenem-resistant *A. baumannii* strains isolated in 2015 [34]. The high colistin resistance rate is most probably related to alterations in the *pmrCAB* operon, as no *mcr* gene was detected in any of the isolates tested and could be ascribed to increased colistin consumption in Greece, due to limited therapeutic options against *A.baumannii*.

**Fig. 2:**
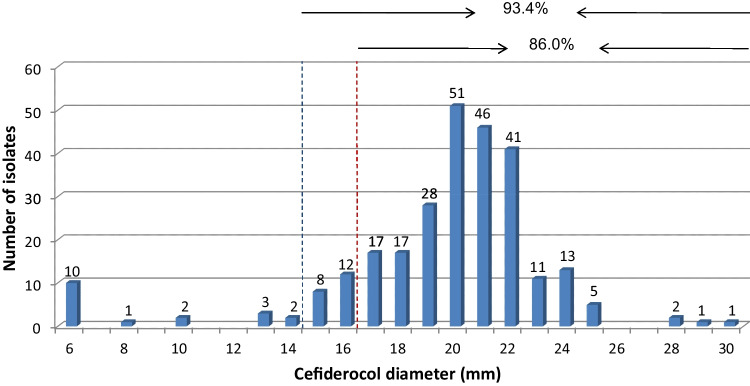
Cefiderocol 30μg disk diameter distribution. The dashed line in blue represents the CLSI susceptibility breakpoint, and the dashed line in red separates the zone diameters of ≥17 mm, which according to EUCAST, correspond to MIC values below the PK-PD breakpoint of S ≤ 2 mg/L

**Fig. 3:**
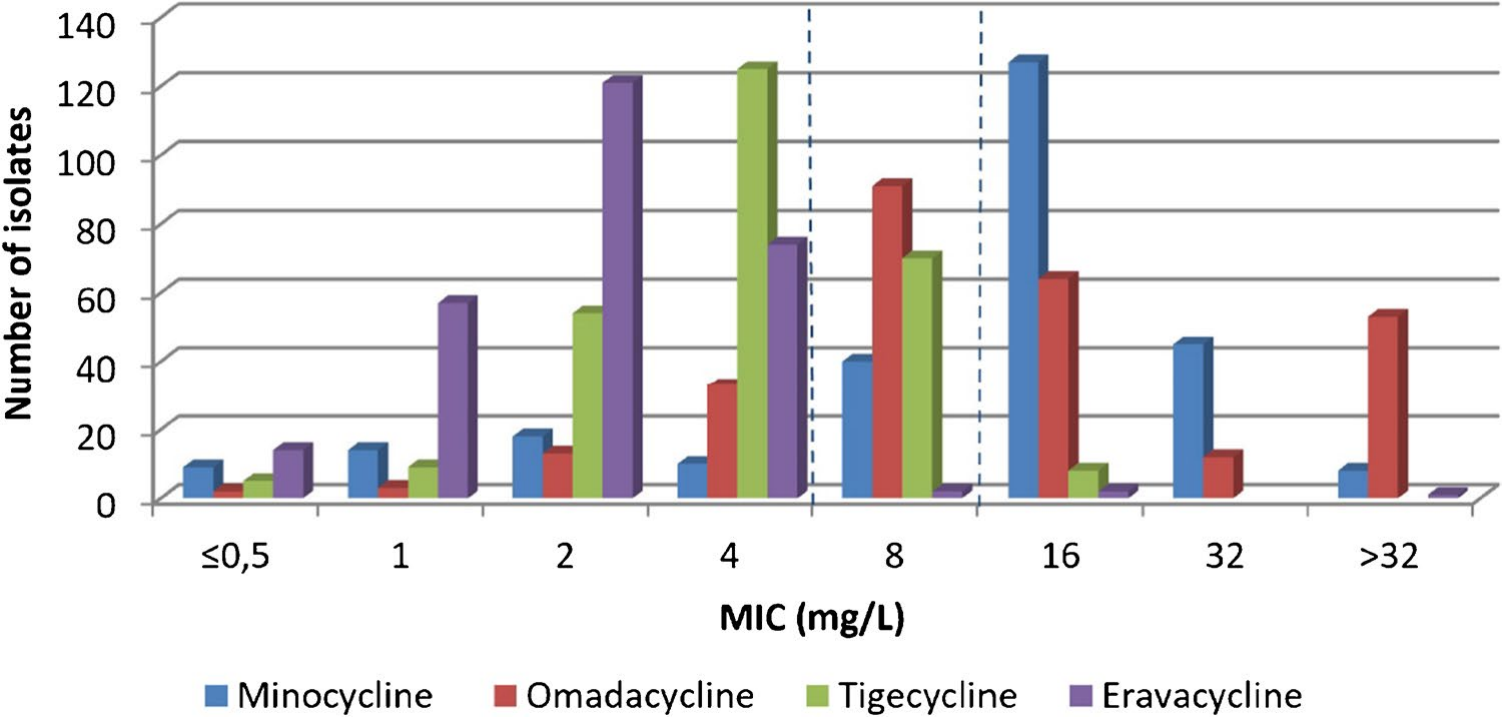
Third-generation tetracyclines MIC distributions, compared to minocycline. The dashed lines represent the CLSI clinical breakpoints for minocycline (S ≤ 4 mg/L; R ≥ 16 mg/L)

In our study, the recently developed, FDA / EMA - approved cefiderocol was active against 86.0% or 93.4% of all *A. baumannii* isolates according to EUCAST or CLSI susceptibility breakpoints respectively (Table 1, Fig. 2). This is consistent with published reports of a susceptibility rate of approximately 94% per CLSI criteria for CRAb isolates originating from North America and Europe [35]. In spite of its documented high level of potency, clinical data do not yet support widespread use for patients with *A. baumannii* infections [36]. It is currently mainly being used for salvage therapy, administered with or without other in vitro active agents, but has not yet been introduced in clinical practice in Greece. This means that local *Acinetobacter* populations have so far been completely unaffected by any sort of impact this agent might exert; raising concern as to the importance of the resistance rate found herein (6.6% per CLSI /14% per EUCAST criteria). Eravacycline MIC values, in this study, ranged from 0.06 to >32mg/L, with MIC_50_/_90_ values of 2/4 mg/L (Table 1, Suppl. Table 2, Fig. 3). This also recently developed and FDA/EMA-approved agent was 8-fold more active than minocycline and 2-fold more active than tigecycline by MIC_50_ /_90_ value comparison, which were in accordance with data from a single-center study in Greece with 100 XDR or PDR *A. baumannii* isolated in 2021 [37] and a worldwide study conducted in 2015-2017[38]. Increased eravacycline MIC values have been associated with increased expression of the AdeABC efflux pump [36].

Lastly, omadacycline MICs ranged between 0.12 and >32, with 129 isolates (47.6%) exhibiting MICs ≥16 (MIC_50_ 8: mg/L) and 53 isolates (19.6%) exhibiting MICs >32 (MIC_90_>32mg/L) (Table 1, Suppl. Table S2, Fig. 3).

**Fig. 4:**
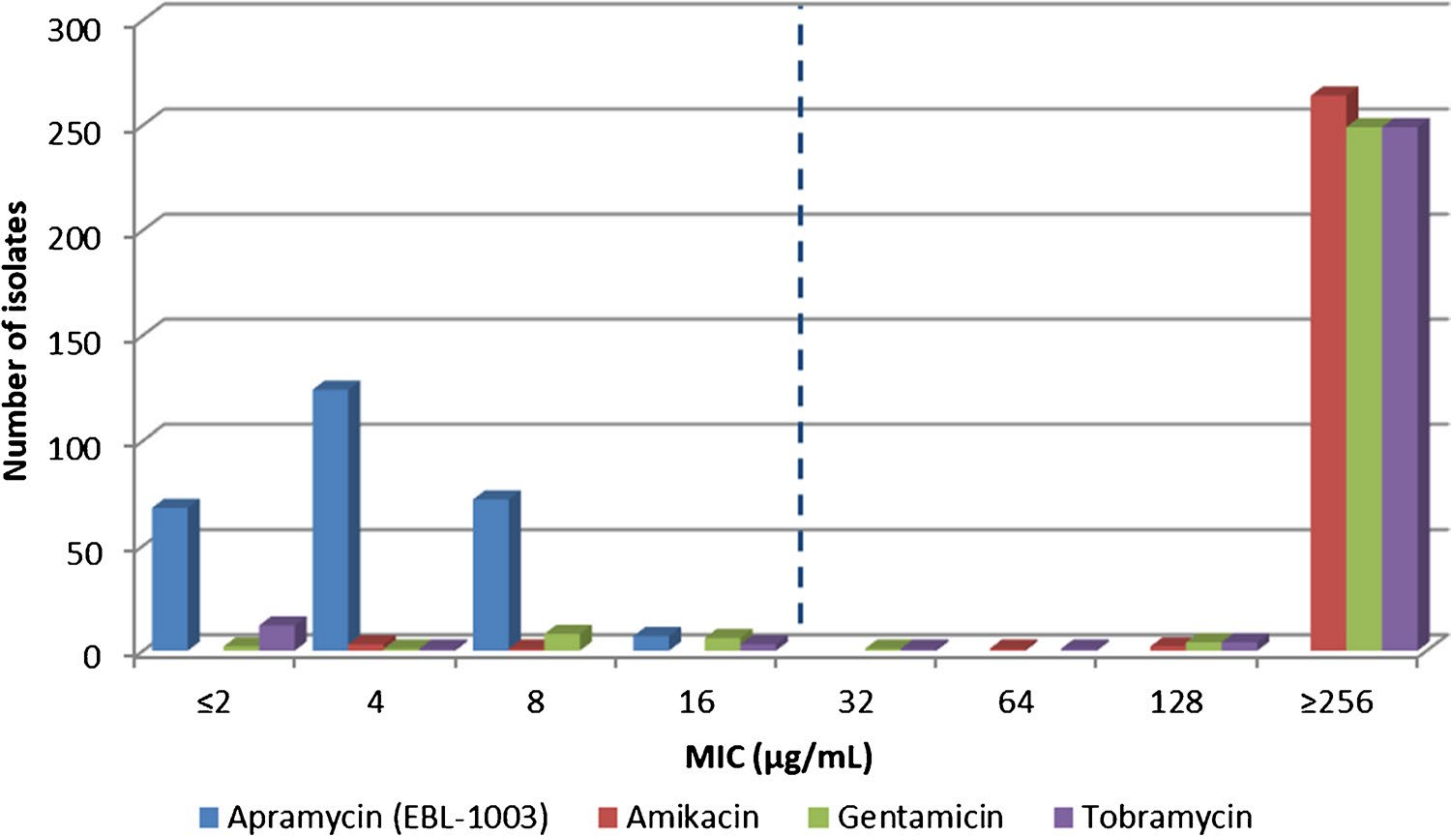
Aminoglycosides MIC distribution. The dashed line represents the preliminary apramycin (EBL-1003) epidemiological cut-off value (ECOFF) [13]

A noteworthy finding of our study was the in vitro activity of the crystalline free base of apramycin (EBL-1003). Its structural distinction has been demonstrated to evade almost all aminoglycoside-resistance mechanisms of clinical relevance, including methylation of N7 at ribosomal site G1405 by 16S rRNA methyltransferases that inactivates the whole range of the 4,6-disubstituted deoxystreptamines [13]. All isolates in this study exhibited an MIC of ≤ 16mg/L (MIC_50_/_90_ 4 / 8 mg/L) suggesting 100% susceptibility according to the preliminary ECOFF (16mg/L) defined by Juhas et al for *A. baumannii* (Fig. 4, Table 1, Suppl. Table S2) [13]. This is consistent with previous reports, which showed that the vast majority of the analyzed *A. baumannii* clinical isolates from Europe, Asia, Africa and South America were more susceptible to apramycin than to other aminoglycosides [13, 31, 39, 40]. Based on the high susceptibility rates, the bactericidal activity reported in a neutropenic murine thigh infection model [41], and its low toxicity [42], apramycin (EBL-1003) may represent a promising next-generation aminoglycoside for the treatment of MDR Gram-negative systemic infections in Greece and elsewhere.

Regarding possible limitations, this study has not utilized whole genome sequencing nor multilocus sequence typing to further elucidate resistance mechanisms to various antimicrobials (i.e., colistin, minocycline, etc.), or to determine *armA* location. On a different note, there was no evaluation of the novel combination agent of sulbactam-durlobactam (SUL-DUR), the new member of the diazabicyclooctane class of β-lactamase inhibitors, with broad spectrum activity against Ambler class A, C and D serine β-lactamases, resulting in the restoration of CR*Ab* isolates susceptibility to β-lactams [43] which appears promising in in vitro studies in Greece [34]. Another possible limitation is the evaluation of cefiderocol activity by disk diffusion and not by MIC determination. On August 18^th^, 2022, EUCAST published a warning against all commercially available MIC determination tests and recommended, cefiderocol testing by disk diffusion until confirmatory MIC determination issues are resolved. When correctly performed and calibrated using quality material and recommended quality control guidelines, disk diffusion adequately predicts susceptibility as zone diameters of ≥17 mm for the cefiderocol 30 μg disk corresponds to MIC values below the PK-PD breakpoint of S ≤ 2 mg/L [17].

Overall, our findings highlight the continued importance of CR*Ab* as a health care-associated pathogen with limited treatment options. CR*Ab* isolates causing infections in Greek hospitals almost exclusively produce OXA-23, the vast majority co-produce the ArmA methyltransferase and belong mainly to IC II. From a clinical point of view, despite its possible limitations, this study importantly illustrates the *in vitro* activities of three novel and one experimental agent against contemporary blood *A. baumannii* isolates. In vitro activity of older colistin appears to have dramatically decreased, while eravacycline seems unable to become the game changer for the treatment of *A. baumannii* infections. Cefiderocol demonstrated potent in vitro activity inhibiting more than 86% of the isolates in this multicenter study, supporting the necessity of further studies to elucidate the role of cefiderocol against *A. baumannii* infections. Finally, the highlight of this study was the promising in vitro activity verified for apramycin (EBL-1003) against this very difficult-to-treat isolate collection, a result that warrants further evaluation for the use of apramycin in the treatment of XDR or PDR *A. baumannii* infections.

## Supplementary information

ESM 1 (DOCX 39 kb)

## Data Availability

The datasets generated during and/or analyzed during the current study are available from the corresponding author on reasonable request.
