# Supplementary material for: In vitro activities of omadacycline, eravacycline, cefiderocol, apramycin, and comparator antibiotics against Acinetobacter baumannii causing bloodstream infections in Greece, 2020–2021: a multicenter study
Source: Eur J Clin Microbiol Infect Dis. 2023 May 3;42(7):843–52. doi: 10.1007/s10096-023-04616-7 (PMC10155660; doi:10.1007/s10096-023-04616-7)
Supplement: Supplementary file 1 — (DOCX 39 kb) [file 10096_2023_4616_MOESM1_ESM.docx]

**Supplementary Table S1. Sequences of primers used for simplex and multiplexPCRs for detection of genes encoding OXA-type carbapenemases,** class B metallo-β-lactamases, **16S rRNA methyltransferases, aminoglycoside modifying enzymes, for detection of *mcr-1*to *mcr-5* genes and for**sequence group typing**in**A.baumannii

| **Gene** | **Primer** | **Sequence (5' → 3')** | **PCR product, bp** | **Reference** |
| --- | --- | --- | --- | --- |
| ***Multiplex PCR for OXA-type catbapenemase genes*** | | | | |
| *bla*_OXA-23_-like | OXA-23F | GATCGGATTGGAGAACCAGA | 501 | 1 |
|  | OXA-23R | ATTTCTGACCGCATTTCCAT |  |  |
| *bla*_OXA-24_-like | OXA-24F | GGTTAGTTGGCCCCCTTAAA | 246 |  |
|  | OXA-24R | AGTTGAGCGAAAAGGGGATT |  |  |
| *bla*_OXA-51_-like | OXA-51F | TAATGCTTTGATCGGCCTTG | 353 |  |
|  | OXA-51R | TGGATTGCACTTCATCTTGG |  |  |
| *bla*_OXA-58_-like | OXA-58F | AAGTATTGGGGCTTGTGCTG | 599 |  |
|  | OXA-58R | CCCCTCTGCGCTCTACATAC |  |  |
| *bla*_OXA-143_-like | OXA-143-F | TGGCACTTTCAGCAGTTCCT | 149 | 2 |
|  | OXA-143-R | TAATCTTGAGGGGGCCAACC |  |  |
| bla_OXA-235_ -like | OXA-235_F | TTGTTGCCTTTACTTAGTTGC | 768 | 3 |
|  | OXA-235_R | CAAAATTTTAAGACGGATCG |  |  |
| ***Simplex PCR for class B metallo-β-lactamase genes*** | | | | |
| bla_VIM_ – type | VIM-F | ATGGTGTTTGGTCGCATATC | 509 | 4 |
|  | VIM-R | TGGGCCATTCAGCCAGATC |  |  |
| bla_NDM_ - type | NDM-F | GAAGCTGAGCACCGCATTAG | 758 | 5 |
|  | NDM-R | GGGCCGTATGAGTGA |  |  |
| *bla*_IMP_ - variants | IMP-F | CTACCGCAGCAGAGTCTTTG | 587 | 6 |
|  | IMP-R | AACCAGTTTTGCCTTACCAT |  |  |
| ***Multiplex PCR for 16S rRNA-methyltransferase genes*** | | | | |
| *rmtA* | rmtAF | AAACTATTCCGCATGGTTC | 88 | 7 |
|  | rmtAR | TCATGTACACAAGCTCTTTCC |  |  |
| *rmtC* | rmtCF | CAGGGGTTCCAACAAGT | 246 |  |
|  | rmtCR | AGAGTATATAGCTTGAACATAAGTAGA |  |  |
| *rmtD* | rmtDF | TCGTTTCAGCACGTAAAACA | 652 |  |
|  | rmtDR | CAGCGCGAAATTCAAAAAGG |  |  |
| *rmtG* | rmtGF | ACGGAATGCCGCGCGAAGTA | 381 |  |
|  | rmtGR | TCTCCGCAAGCAGATCGCCG |  |  |
| *rmtH* | rmtHF | ATGACCATTGAACAGGCAGC | 464 |  |
|  | rmtHR | AGGGCAAAGGTAAAATCCCA |  |  |
| *armA* | armAF | ATTTTAGATTTTGGTTGTGGC | 101 |  |
|  | armAR | ATCTCAGCTCTATCAATATCG |  |  |
| *npmA* | npmAF | GGGCTATCTAATGTGGTG | 229 |  |
|  | npmAR | TTTTTATTTCCGCTTCTTCGT |  |  |
| *rmtB* | rmtBF | ACTTTTACAATCCCTCAATAC | 171 |  |
|  | rmtBR | AAGTATATAAGTTCTGTTCCG |  |  |
| *rmtE* | rmtEF | GATGCCGTGTCTGTTACGCCG | 446 |  |
|  | rmtER | ACGTGAACCCACGAGTCCTGC |  |  |
| *rmtF* | rmtFF | CGATCCTACTGGGCTCCAT | 314 |  |
|  | rmtFR | GGCATAGTGCTTTTCCATGC |  |  |
| ***Simplex PCR for armA (confirmation)*** | | | | |
| *armA* | armA-F | ATTCTGCCTATCCTAATTGG | 315 | 8 |
|  | armA-R | ACCTATACTTTATCGTCGTC |  |  |
| ***Multiplex PCR for mcr-variants*** | | | | |
| mcr-1 | mcr1*_*320bp_fw | AGTCCGTTTGTTCTTGTGGC | 320 | 9 |
|  | mcr1_320bp_rev | AGATCCTTGGTCTCGGCTTG |  |  |
| mcr-2 | mcr2*_*700bp_fw | CAAGTGTGTTGGTCGCAGTT | 715 |  |
|  | mcr2*_*700bp_rev | TCTAGCCCGACAAGCATACC |  |  |
| mcr-3 | mcr3*_*900bp_fw | AAATAAAAATTGTTCCGCTTATG | 929 |  |
|  | mcr3*_*900bp_rev | AATGGAGATCCCCGTTTTT |  |  |
| mcr-4 | mcr4*_*1100bp_fw | TCACTTTCATCACTGCGTTG | 1.116 |  |
|  | mcr4*_*1100bp_rev | TTGGTCCATGACTACCAATG |  |  |
| mcr-5 | MCR5*_*fw | ATGCGGTTGTCTGCATTTATC | 1.644 |  |
|  | MCR5*_*rev | TCATTGTGGTTGTCCTTTTCTG |  |  |
| ***Simplex PCR for genes encoding for aminoglycoside modifying enzymes*** | | | | |
| *aph(3')-VI* | APH(3')-VIF | CGGAAACAGCGTTTTAGA | 716 | 10 |
|  | APH(3')-VIR | TTCCTTTTGTCAGGTC |  |  |
| aac(6’)-Ib | AAC(6’)-IF | TATGAGTGGCTAAATCGΑT | 395 | 11 |
|  | AAC(6’)-IR | CCCGCTTTCTCGTAGCA |  |  |
| aac(3’)-Ia | AAC(3’)-IF | ACAAAGTTAGGTGGCTCAAGTATGGGCATC | 407 | 12 |
|  | AAC(3’)-IR | TCACCGTAATCTGCTTGCACGTAGATCAC |  |  |
| aac(3’)-IV | AAC(3’)-IVF | CTCGAAGATGGGCCACTTGGACTGATC | 367 | 12 |
|  | AAC(3’)-IVR | AACTCGGCAAGATGCAGCGTCGTG |  |  |
| ant(2’)-Ia | ANT(2’)-IF | ATGCGCTCACGCAACTGGTC | 749 | 13 |
|  | ANT(2’)-IR | GCATATCGCGACCTGAAAGC |  |  |
| ***Trilocus multiplex PCRs for sequence group typing*** | | | | |
| Group 1  *ompA* | Group1ompAF306 | GATGGCGTAAATCGTGGTA | 355 | 14 |
|  | Group1and2ompAR660 | CAACTTTAGCGATTTCTGG |  |  |
| Group 1  *csuE* | Group1csuEF | CTTTAGCAAACATGACCTACC | 702 |  |
|  | Group1csuER | TACACCCGGGTTAATCGT |  |  |
| Group 1  *bla*_OXA-51_-like | Gp1OXA66F89 | GCGCTTCAAAATCTGATGTA | 559 |  |
|  | Gp1OXA66R647 | GCGTATATTTTGTTTCCATTC |  |  |
| Group 2  *ompA* | Group2ompAF378 | GACCTTTCTTATCACAACGA | 343 |  |
|  | Group1and2ompAR660 | CAACTTTAGCGATTTCTGG |  |  |
| Group 2  *csuE* | Group2csuEF | GGCGAACATGACCTATTT | 580 |  |
|  | Group2csuER | CTTCATGGCTCGTTGGTT |  |  |
| Group 2  *bla*_OXA-51_-like | Gp2OXA69F169 | CATCAAGGTCAAACTCAA | 162 |  |
|  | Gp2OXA69R330 | TAGCCTTTTTTCCCCATC |  |  |

**Supplementary Table S2.** MIC and ‘cumulative percentage inhibited’ distributions, MIC_50_ and MIC_90_ values and antimicrobial susceptibilities of 271 *Acinetobacter baumannii* isolates.

| Antimicrobial agent | No. (cumulative %) of isolates inhibited at MIC (mg/L) | | | | | | | | | | MIC_50_ (mg/L) | MIC_90_ (mg/L) | Susceptibility^a^ (%) |
| --- | --- | --- | --- | --- | --- | --- | --- | --- | --- | --- | --- | --- | --- |
|  | ≤0.5 | 1 | 2 | 4 | 8 | 16 | 32 | 64 | 128 | ≥256 |  |  |  |
| Apramycin  (EBL-1003) |  |  | 68^b^  (25.09) | 124  (70.85) | 72  (97.42) | 7  (100) |  |  |  |  | 4 | 8 | ND |
| Amikacin |  |  |  | 3  (1.11) | 1  (1.48) |  |  | 1  (1.85) | 2  (2.58) | 264  (100) | ≥256 | ≥256 | 1.48 |
| Gentamicin |  |  | 2^b^  (0.74) | 1  (1.11) | 8  (4.06) | 6  (6.27) | 1  (6.64) |  | 4  (8.12) | 249  (100) | ≥256 | ≥256 | 1.11 |
| Tobramycin |  |  | 12^b^  (4.43) | 1  (4.80) |  | 3  (5.90) | 1  (6.27) | 1  (6.64) | 4  (8.12) | 249  (100) | ≥256 | ≥256 | 4.80 |
| Imipenem | 1  (0.37) |  | 15  (5.90) | 26  (15.50) | 66  (39.85) | 115  (82.29) | 26  (91.88) | 22^c^  (100) |  |  | 16 | 32 | 5.90 |
| Meropenem | 2  (0.74) |  | 1  (1.11) |  |  | 33  (13.28) | 108  (53.14) | 127^c^  (100) |  |  | 32 | >32 | 1.11 |
| Minocycline | 9  (3.32) | 14  (8.49) | 18 (15.13) | 10  (18.82) | 40  (33.58) | 127  (80.44) | 45  (97.05) | 8^c^  (100) |  |  | 16 | 32 | 18.8^d^ |
| Tigecycline | 5  (1.85) | 9  (5.17) | 54 (25.09) | 125  (71.22) | 70  (97.05) | 8  (100) |  |  |  |  | 4 | 8 | ND |
| Eravacycline | 14  (5.17) | 57  (26.20) | 121 (70.85) | 74  (98.15) | 2  (98.89) | 2  (99.63) |  | 1^c^  (100) |  |  | 2 | 4 | ND |
| Omadacycline | 2  (0.74) | 3  (1.85) | 13 (6.64) | 33  (18.82) | 91  (52.40) | 64  (76.01) | 12  (80.44) | 53^c^  (100) |  |  | 8 | >32 | ND |
| Colistin | 3  (1.11) | 16  (7.01) | 23  (15.50) | 18  (22.14) | 48  (39.85) | 40  (54.61) | 48  (72.32) | 75^c^  (100) |  |  | 16 | >32 | 15.50 |
| Trimethoprim-sulfamethoxazole^e^ | 1  (0.37) | 1  (0.74) | 3  (1.85) | 6  (4.06) | 5  (5.90) | 5  (7.75) | 9  (11.07) | 241^c^  (100) |  |  | >32 | >32 | 1.85 |

ND, not determined; ^a^, according to EUCAST and CLSI breakpoints; ^b^, ≤2mg/L; ^c^, >32mg/L; ^d^, according to CLSI breakpoints; ^e^, Trimethoprim-sulfamethoxazole MICs are expressed as the trimethoprim concentration.

**References**

1. Woodford N, Ellington MJ, Coelho JM, Turton JF, Ward ME, Brown S, Amyes SG, Livermore DM (2006) Multiplex PCR for genes encoding prevalent OXA carbapenemases in *Acinetobacter* spp. Int J Antimicrob Agents 27: 351-3. <https://doi.org/10.1016/j.ijantimicag.2006.01.004>
2. Higgins PG, Lehmann M, Seifert H (2010) Inclusion of OXA-143 primers in a multiplex polymerase chain reaction (PCR) for genes encoding prevalent OXA carbapenemases in *Acinetobacter* spp. Int J Antimicrob Agents 35: 305. <https://doi.org/10.1016/j.ijantimicag.2009.10.014>
3. Higgins PG, Pérez-Llarena FJ, Zander E, Fernández A, Bou G, Seifert H (2013) OXA-235, a novel class D β-lactamase involved in resistance to carbapenems in *Acinetobacter baumannii*. Antimicrob Agents Chemother 57: 2121-6. <https://doi.org/10.1128/AAC.02413-12>
4. Poirel L, Naas T, Nicolas D, Collet L, Bellais S, Cavallo JD, Nordmann P (2000) Characterization of VIM-2, a carbapenem-hydrolyzing metallo-beta-lactamase and its plasmid- and integron-borne gene from a *Pseudomonas aeruginosa* clinical isolate in France. Antimicrob Agents Chemother 44: 891-7. <https://doi.org/10.1128/AAC.44.4.891-897.2000>
5. Sidjabat H, Nimmo GR, Walsh TR, Binotto E, Htin A, Hayashi Y, Li J, Nation RL, George N, Paterson DL (2011) Carbapenem resistance in *Klebsiella pneumoniae* due to the New Delhi Metallo-β-lactamase. Clin Infect Dis 52: 481-4. <https://doi.org/10.1093/cid/ciq178>
6. Senda K, Arakawa Y, Ichiyama S, Nakashima K, Ito H, Ohsuka S, Shimokata K, Kato N, Ohta M (1996) PCR detection of metallo-β-lactamase gene (*bla*_IMP_) in gram-negative rods resistant to broad-spectrum β-lactams. J Clin Microbiol 34: 2909–2913. <https://doi.org/10.1128/jcm.34.12.2909-2913.1996>
7. Corrêa LL, Montezzi LF, Bonelli RR, Moreira BM, Picão RC (2014) Revised and updated multiplex PCR, targeting acquired 16S rRNA methyltransferases. Int J Antimicrob Agents 43:479–481. <https://doi.org/10.1016/j.ijantimicag.2014.02.003>
8. Doi Y, Arakawa Y (2007) 16S ribosomal RNA methylation: emerging resistance mechanism against aminoglycosides. Clin Infect Dis45:88-94. https://doi.org/10.1086/518605
9. Rebelo AR, Bortolaia V, Kjeldgaard JS, Pedersen SK, Leekitcharoenphon P, Hansen IM, Guerra B, Malorny B, Borowiak M, Hammerl JA, Battisti A, Franco A, Alba P, Perrin-Guyomard A, Granier SA, De Frutos Escobar C, Malhotra-Kumar S, Villa L, Carattoli A, Hendriksen RS (2018) Multiplex PCR for detection of plasmid-mediated colistin resistance determinants, *mcr-1, mcr-2, mcr-3, mcr-4 and mcr-5* for surveillance purposes. Euro Surveill 23: 17–00672. <https://doi.org/10.2807/1560-7917.ES.2018.23.6.17-00672>
10. Noppe-Leclercq I, Wallet F, Haentjens S, Courcol R, Simonet M (1999) PCR detection of aminoglycoside resistance genes: a rapid molecular typing method for*Acinetobacter baumannii.* Res Microbiol; 150: 317-22. <https://doi.org/10.1016/s0923-2508(99)80057-6>
11. Ploy MC, Giamarellou H, Bourlioux P, Courvalin P, Lambert T (1994) Detection of *aac(6')-I* genes in amikacin-resistant *Acinetobacter spp*. by PCR. Antimicrob Agents Chemother 38: 2925-8. <https://doi.org/10.1128/AAC.38.12.2925>
12. Aggen JB, Armstrong ES, Goldblum AA, Dozzo P, Linsell MS, Gliedt MJ, Hildebrandt DJ, Feeney LA, Kubo A, Matias RD, Lopez S, Gomez M, Wlasichuk KB, Diokno R, Miller GH, Moser HE (2010) Synthesis and spectrum of the neoglycoside ACHN-490. Antimicrob Agents Chemother 54: 4636-42. <https://doi.org/10.1128/AAC.00572-10>
13. Dubois V, Arpin C, Dupart V, Scavelli A, Coulange L, André C, Fischer I, Grobost F, Brochet JP, Lagrange I, Dutilh B, Jullin J, Noury P, Larribet G, Quentin C (2008) Beta-lactam and aminoglycoside resistance rates and mechanisms among *Pseudomonas aeruginosa* in French general practice (community and private healthcare centres). J Antimicrob Chemother 62: 316-23. <https://doi.org/10.1093/jac/dkn174>
14. Turton JF, Gabriel SN, Valderrey C, Kaufmann ME, Pitt TL (2007) Use of sequence-based typing and multiplex PCR to identify clonal lineages of outbreak strains of *Acinetobacter baumannii*. Clin Microbiol Infect 13: 807-15. <https://doi.org/10.1111/j.1469-0691.2007.01759.x>
